# Molecular Approaches To Target GPCRs in Cancer Therapy

**DOI:** 10.3390/ph4040567

**Published:** 2011-03-25

**Authors:** Giulio Innamorati, Maria Teresa Valenti, Francesco Giovinazzo, Luca Dalle Carbonare, Marco Parenti, Claudio Bassi

**Affiliations:** 1 L.U.R.M. University Laboratory for Medical Research, University of Verona, Piazzale L.A. Scuro 10, Verona 37134, Italy; 2 Department of Pathology and Diagnostic, G.B. Rossi Hospital, University of Verona, Piazzale L.A. Scuro 10, Verona 37134, Italy; 3 Department of Medicine, G.B. Rossi Hospital, University of Verona, Piazzale L.A. Scuro 10, Verona 37134, Italy; 4 Department of Surgery, G.B. Rossi Hospital, University of Verona, Piazzale L.A. Scuro 10, Verona 37134, Italy; 5 Department of Experimental Medicine, University of Milano-Bicocca, Monza 20052, Italy

**Keywords:** molecular pharmacology, cancer, G protein coupled receptor, heterotrimeric G protein

## Abstract

Hundreds of G protein coupled receptor (GPCR) isotypes integrate and coordinate the function of individual cells mediating signaling between different organs in our bodies. As an aberration of the normal relationships that organize cells' coexistence, cancer has to deceive cell-cell communication in order to grow and spread. GPCRs play a critical role in this process. Despite the fact that GPCRs represent one of the most common drug targets, current medical practice includes only a few anticancer compounds directly acting on their signaling. Many approaches can be envisaged to target GPCRs involved in oncology. Beyond interfering with GPCRs signaling by using agonists or antagonists to prevent cell proliferation, favor apoptosis, induce maturation, prevent migration, *etc.*, the high specificity of the interaction between the receptors and their ligands can be exploited to deliver toxins, antineoplastic drugs or isotopes to transformed cells. In this review we describe the strategies that are in use, or appear promising, to act directly on GPCRs in the fight against neoplastic transformation and tumor progression.

## 1. Introduction

Since their appearance in evolution, nature has evolved thousands of G protein coupled receptor (GPCR) isoforms to operate as cellular sensors in the most diversified organisms populating the earth. The variety of stimuli acting on GPCRs includes photons, lipids, peptides, amino acids, ions, *etc.* The recognition of the stimulus occurs with an extraordinarily high specificity. Information about the presence, and even the direction from where the stimulation originated, is efficiently relayed in spite the interaction may occur at broad ranges of concentrations (from below picomolar to above millimolar) or even with the ligand tethered to the surface of other cells (like in the cases of fractalkine/CX3CL1 [1] and CXCL16 [2]).

GPCRs are therefore extremely versatile tools and represent a great evolutionary success. All share the same seven transmembrane domains structure and their signaling converges on common downstream effectors and modulators (such as G proteins, arrestins, GPCRs kinases/GRKs). In multicellular organisms, GPCRs became indispensable to integrate and coordinate the function and proliferation of individual cell types. As an aberration of the normal relationships that organize cells coexistence, tumors commonly deceive cell-cell communication in order to expand and spread in the body. GPCRs represent critical elements in this processes too [3]. A very recent genomic characterization (1,507 coding genes from 441 tumors) of somatic mutations within the cancer genomes of multiple cancer types revealed an underestimated role for G proteins signaling [4].

Despite GPCRs represent one of the major pharmaceutical targets; it is surprising that the clinical practice of cancer treatment includes only a few drugs that act on GPCR-mediated signaling. Among the sporadic examples is the gold standard of endocrine treatment for hormone responsive prostate and breast cancers. Progression and growth of prostate cancer cells require the production of testosterone via a signaling cascade that begins with the secretion of gonadotropin releasing-hormone (GnRH) from the hypothalamus. GnRH subsequently induces synthesis and secretion of two GPCR agonists from the pituitary gland: luteinizing hormone (LH) and follicle-stimulating hormone (FSH). As a result, steroidogenesis is induced in adrenal glands and testes. Testosterone is next released and reaches the prostate where it stimulates cancer cells growth. Molecules acting on GnRH receptor (see Sections 2 and 4) are thus used to indirectly reduce testosterone levels.

Two other GPCRs ligands are prescribed for cancer treatment, octreotide and pegvisomant. Octreotide is a synthetic somatostatin (SST) agonist. SST inhibits the pituitary gland to secrete growth hormone (GH) and insulin-like growth factor 1 (IGF-1) [5]. GH antagonists and SST agonists are highly effective antiproliferative drugs. Octreotide prevents over-production of GH by pituitary somatotroph adenomas associated with acromegaly. In addition, octreotide is utilized to counteract the effects of SST secreting malignant gastroenteropatic neuroendocrine tumors. More recently, a cyclohexapeptide analogue of octreotide was developed (pasireotide) that binds to a larger number of SST receptor isotypes, hence more closely mimicking the action of the natural ligands. Pegvisomant, is a pegylated peptide acting as GH antagonist licensed as a third or fourth line option when other treatments have failed to normalize IGF-1 levels.

Both approaches described above act indirectly to inhibit cell growth or to prevent secondary effects caused by peptides released from the tumor. However, there is a wealth of opportunities for directly targeting GPCRs expressed on tumor cells.

**Figure 1:**
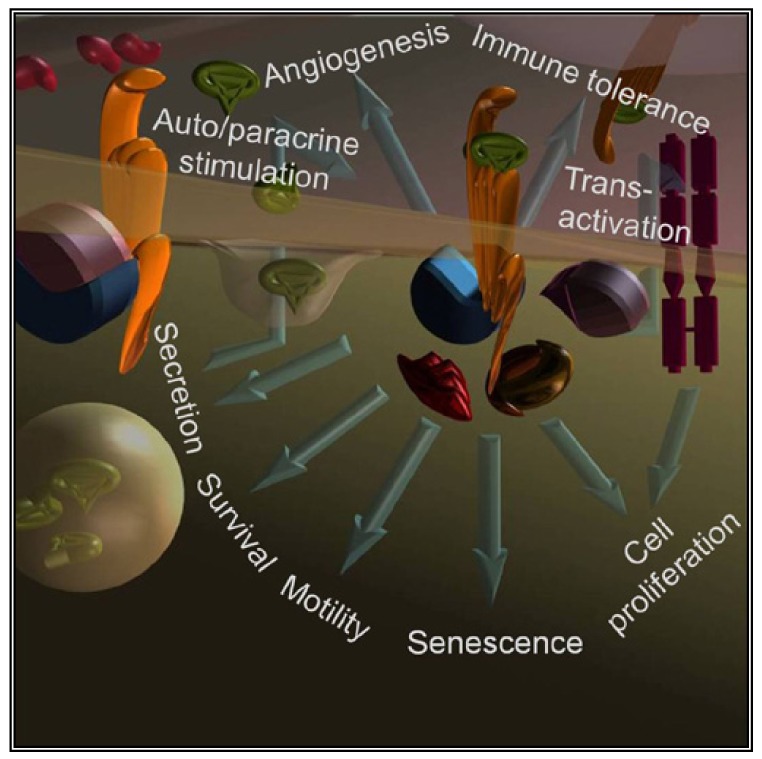
Receptor (yellow) occupancy by the agonist (green) promotes the exchange of guanosine diphosphate (GDP) for guanosine triphosphate (GTP) inducing the activation of the heterotrimeric G protein. The α (blue) and the βγ (pink) subunits separate to transmit the signal toward downstream effectors leading to a large number of physiological responses. GPCRs-mediated autocrine and paracrine loops in cancer cells have been described in a variety of malignancies. In some instances, the signaling pathway may imply the activation of other membrane receptors. After entering the cell via GPCRs, the signal may exit to re-enter via RTKs (magenta). This process has been termed transactivation and occurs due to as yet poorly identified mechanisms that activate extracellular proteases to shed plasma membrane-bound growth factor precursors. GPCR signaling may also be directed to effectors other than the G proteins. The best described is arrestin (in red), the adaptor protein initially recognized as an inhibitor of GPCR activity. Other signaling intermediates activated by direct interaction with the GPCR include signal transducer and activator of transcription (STAT) family members (brown).

Abnormal expression of GPCRs and/or their ligands is directly observed in cancer cells of various origins that abuse GPCRs signaling to directly stimulate growth, induce angiogenesis, inhibit apoptosis, promote spreading and induce immune-tolerance [3,6] (Figure1).

In lung, gastric, colorectal, pancreatic and prostatic cancers, sustained GPCRs stimulation is promoted by activatory autocrine and paracrine loops [7,8] created by specific neuropeptides with well-defined physiologic actions (such as neurotransmitters and gut hormones). In addition, the tumor microenvironment may provide an abundant source of GPCRs stimulants, as in the case of chemokines produced by chronic inflammatory phenomena [9]. The same cancer cells may shape the microenvironment to favor an immunosuppressive environment by secreting chemokines that alter the composition of the cells infiltrating the tumor [10].

Furthermore, a tight operational relationship occurs between GPCRs and other receptors responding to growth factors. GPCRs signaling may precede, follow, parallel or synergize the signaling of receptors for steroids, epidermal growth factor (EGF), platelet derived growth factor (PDGF), *etc.* The GPCR GPR30 synergizes classic estrogen receptors ERα and β responding to the same mitogenic stimulus [11]. Receptor tyrosin kinases (RTKs) are under transcriptional regulation of GPCRs and *vice versa* [12]. In addition, GPCRs can be upstream (see signaling by β2 or α1a adrenergic [13-15], chemokines [16], lysophosphatidic acid/LPA [17], CXCL1 [18] and sphingosine 1-phosphate/S1P [19], endothelin 1 [20] receptors) or downstream (S1P [21] receptor) the activation of RTKs (EGF, PDGF, IGF1, nerve growth factor (NGF), vascular endothelial growth factor (VEGF), hepatocyte growth factor (HGF) receptors [12]) in a phenomenon defined transactivation (Figure1). Transactivation and intracellular crosstalks between GPCRs and RTKs are often determinant in explaining how GPCRs signal to ERK and hence promote cell growth/proliferation as reviewed in details [22,23].

In this review we describe the strategies that can be exploited to directly act on GPCRs to oppose neoplastic transformation and tumor progression.

## 2. Antagonists

**Figure 2:**
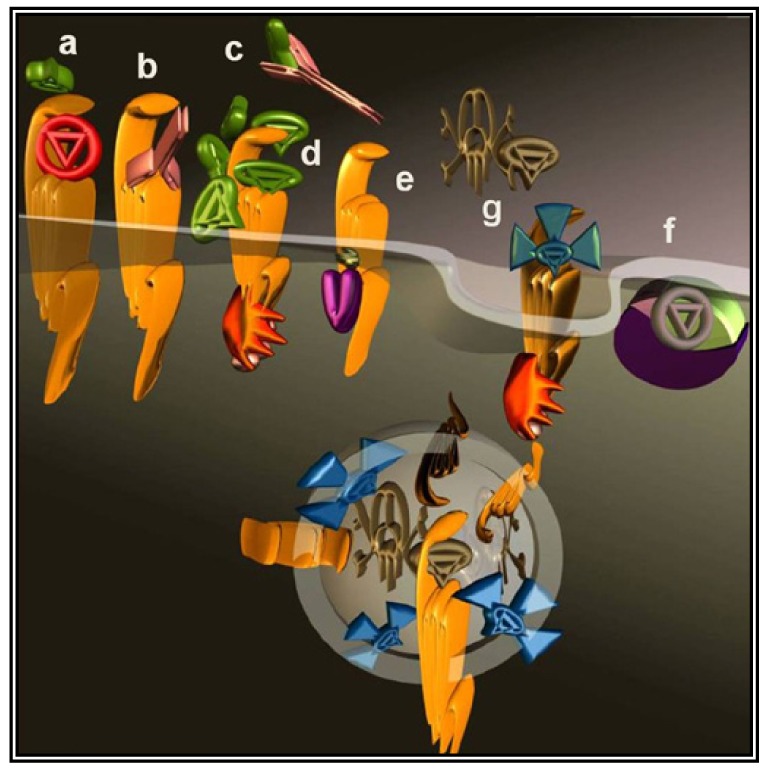
GPCR (yellow) signaling can be inhibited by different approaches. (**a**). Antagonists are small molecules (red) capable of antagonizing the interaction of the endogenous agonist (green); (**b**). Antibodies (pink) can act either masking the agonist binding site; (**c**). Or binding and quenching the agonist itself; (**d**). Sustained stimulation will also prevent receptor function by promoting arrestin (orange) dependent desensitization and eventually down regulation; (**e**). An acylated GPCR intracellular loop (magenta) can block signaling directed to the G protein by competing with the corresponding GPCR fragment; (**f**). G protein signaling can be directly blocked by specific inhibitors (grey); (**g**). Receptor internalization promotes the endocytosis of radioligands (blue) and toxins (brown) linked to the agonist concentrating cytotoxicity in the endosomal lumen of the cell.

The most intuitive approach to counteract the pro-malignancy effects of GPCR signaling is to use antagonists or inverse agonists (Figure 2a). This approach seems particularly appealing considering the number of specific molecules well-characterized and approved by regulatory agencies for other indications.

As mentioned above, the GnRH receptor is a well established target. Several potent peptide antagonist analogues of GnRH (such as abarelix, acyline, azaline B, cetrorelix, degarelix, ganirelix, iturelix, LXT-101, teverelix, ornirelix and ozarelix) have been clinically tested and several orally active non-peptide antagonists are under development [24]. Furthermore, the GnRH receptor is being evaluated as a target to lower estrogen levels in related proliferative diseases.

Beside the GnRH receptor, a plethora of potential GPCR targets is available and under scrutiny. Endothelin (ET) is considered to promote the growth of many tumors including lung, breast, ovary and prostate cancers [6,25]. The antagonist (ABT-627) has undergone phase II trials for treatment of hormone-resistant prostate cancer as it prevents the protumoral activity of the ET A receptor (ET_a_R). However, atrasentan recognizes with lower affinity also the B isoform of the entothelin receptor (ET_b_R) that conversely has proapoptotic effects. ZD4054 represents a more effective option having higher specificity for the ET_a_R, good tolerability and promising effects on overall survival as compared to placebo. Development is now in phase III [26] and in 2011 an application will be filed having hormone-resistant prostate cancer as initial indication.

Other excellent candidates that could be targeted by antagonists, are GPCRs involved in embryonic signaling pathways that are also crucial to preserve cancer stem cells (CSCs). According to the CSCs hypothesis, only a small fraction of immature cellular intermediates is responsible for mediating tumor expansion, resistance, metastases. Protected by quiescence, the same cells are particularly resistant to pharmacological therapy as they express proteins warranting efficient efflux of xenobiotic toxins (e.g., multidrug-resistant proteins and related members of the ATP-binding cassette [ABC] transporter family). CSC would thus be the tumor component that diffuses out of the organ where it cannot be eradicated by surgical therapy and causes relapses resisting to chemo/radio-therapies [27]. Smoothened (Smo), Frizzled and chemokine receptors are among the receptors that collect signals produced by the environmental niche to preserve staminality and nourish these cellular precursors.

Smo is an unconventional GPCR element of the sonic hedgehog (Hh) cell-signaling pathway that activates the GLI family of transcription factors. Normally active during embryonic development, aberrant reactivation of Hh signaling promotes cancer cell proliferation and survival [28,29]. Cyclopamine and Jervine are two natural alkaloids derived from *Veratrum californicum* that antagonize Smo activity by direct binding to the receptor molecule. Cyclopamine's antitumoral activity was reported in various tumor models and in a phase I study it was effective in inoperable skin cancer patients [30]. Drug discovery produced a number of screening platforms based on biological assays to develop novel Smo antagonists that would mimic cyclopamine with improved drug properties. As extensively reviewed by Peukert *et al.* [31], the majority of the inhibitors of Hh pathways act on Smo (GDC0449, IPI-926, LDE225, PF04449913, XL139) and are now being tested in phase II or phase I clinical trials for the treatment of several advanced tumoral forms [32].

During malignant progression, carcinomas actively remodel the extracellular matrix and tumor stroma creating a favorable microenvironment. In carcinomas, the effect is typically associated to a transition from epithelial to mesenchymal cellular stages as the tumor develops in the primary site and spreads in metastasis. This process appears instrumental to CSC existence [33]. Initially described during normal embryonic development, epithelial to mesenchymal transition (EMT) is determined by a heterogeneous and transient number of mechanisms highly integrated with master developmental and tumorigenic transformation signaling. With RTKs, members of the Frizzled family activate upstream signaling producing EMT. The Wnt/Frizzled pathway is also involved in various differentiation events during embryonic development and, when aberrantly activated, can lead to tumor formation [34]. The activation scheme through which the Wnt ligand produces its effects deviates from the general scheme assumed to apply to GPCRs activation and implies the binding to a receptor complex composed of Frizzled and a co receptor named ‘low-density lipoprotein receptor-related protein’ (LRP). LRP and Frizzled activate signaling that includes classical G protein cascade paralleled by the ‘canonical pathway’ and the planar cell polarity pathway. Frizzled, RTKs and integrins signaling converge inhibiting GSK-3β, a constitutively activated kinase. Preventing GSK-3β action by quenching β-catenin results in the activation of several transforming genes [35]. Aiming to prevent EMT and target CSCs, drugs inhibiting RTKs signaling have been developed (for example, inhibitors of hepatocyte growth factor receptor/c-Met [36]) while several other effectors participating to this complex signaling network, including Frizzled, are studied at the preclinical level [37,38].

CSCs colonize precise microenvironments following cues created by, but not limited to, chemokines such as CXCL12 (stromal derived factor 1/SDF-1) [39]. In normal cell physiology, these gradients form the trails that orient professional migrating cells (leukocytes, embryonic neuroblasts, fibroblasts, *etc.*). Agonists or antagonists may become useful tools to spoil similar paths and thus prevent tumor-initiating cells from finding and colonizing the appropriate niches. Obviously, this approach will be ineffective on tumor masses that are already formed, but it is expected to prevent metastases, a process that accounts for more than 90% of cancer-related deaths and for which to date there is no specifically approved treatment.

As recently reviewed [10,40], several clinical trials (many ongoing) have targeted chemokines receptors, in particular CXCR4, the receptor responding to CXCL12 (SDF-1). CXCR4 has been included among stem cell markers [41] and its importance in tumor development is rapidly emerging [42-44]. Beside promoting cell motility and directing cell migration, CXCR4-mediated multiple signaling pathways promote survival of immature cells and CSCs. Ligands inhibiting CXCR4 (such as plerixafor, BKT140, AMD3100, AMD3465, AMD070, CTCE-9908, RCP168 MSX-122, T22, T140, TN14003, FC131) are under investigation particularly for preventing metastasis [10,45].

The signaling of other chemokines is being thoroughly evaluated. Mostly studied in leukemia, the CCL5-CCR5 axis promotes migration to the bone marrow and osteolysis but it is also active in other solid tumors, most comprehensive findings from breast cancer studies [46]. CCR5 plays a central role in the crosstalk between mesenchymal stem cells and cancer cells. Like CXCR4, CCR5 has received much attention for its involvement in HIV infection. Maraviroc is a CCR5 antagonist recently approved by the FDA for this disease. Approval could open the way to trials testing Maraviroc as an anticancer agent.

In a xenograft model of human breast cancer, the CXCR1 antagonist reparixin was able to specifically target the CSCs population. Reparixin was originally developed to antagonize CXCL8 (IL-8) and thus target CXCR1 and CXCR2 on the surface of neutrophils aiming to prevent their migration to sites of inflammation [47]. A phase I study demonstrated lack of toxicity, however the molecule missed the primary endpoint in phase II trials designed to demonstrate a reduction of tissue damage after myocardial infarction or stroke. Currently, a phase II study for new indications will be initiated after it was demonstrated that reparixin reduced the development of metastasis when administered in mice after injection of breast cancer cells [48]. CXCR2 [49,50] responds to CXCL1 and CXCL8, that are involved in tumor growth, angiogenesis, metastasis and inflammatory infiltration. Various CXCR2 antagonists have been developed (AZD 5122, SB 265610 [51], SB 332235, ICN 197, DF 2156A [52], SCH-479833 and SCH-527123 [53]) and phase II studies have been completed that included cancer among the indications, however, no further developments have been reported so far.

CCR7 expression positively correlates with nodal metastasis and poor prognosis in several cancer forms. CCR7 expression is related to its function in directing cells towards the lymph nodes and in animal models was shown to drive metastasis towards draining lymph nodes. The development of specific antagonists for poorly characterized chemokine receptors (such as CCX754 to target CXCR7 [54]) is thus required to explore new potential targets for preventing metastases.

Preventing autocrine stimulatory loops by an individual antagonist may not apply to all cases since quite often different GPCR isoforms operate redundantly. For example, small cells lung carcinoma (SCLC) is nurtured by multiple simultaneous neuropeptide autocrine systems. To oppose similar signaling and induce apoptosis, a novel class of neuropeptide derivatives based on the substance P sequence have been proposed that exhibit broad specificity for neuropeptide receptors [55]. Personalized cocktails could provide an alternative if setting the appropriate mix would not result too demanding from the diagnostic point of view. However, broadening the spectrum of antagonized ligands will inevitably add up side effects interfering with the physiological signaling, even though, occasionally, shutting off the normal signaling may even result advantageous as in the case of the CCK2 receptor. The CCK2 receptor recognizes gastrin, a potent stimulator of gastric acid secretion and a growth factor for pancreatic, stomach and colorectal cancers. The antagonist Z-360 was evaluated in a phase Ib/IIa study in combination with gemcitabine to treat unresectable pancreas cancer antagonizing gastrin. Z-360 could double benefit the patient by simultaneously suppressing acid secretion and the growth-promoting effects of gastrin [56]. This study showed good tolerability, further studies will demonstrate the effectiveness of combined treatments.

The usage of synthetic antagonists to target GPCRs involved in cancer progression is therefore receiving increasing attention, however other molecular approaches are available to directly intervene on the receptor molecule.

## 3. Vaccine and Neutralizing Antibodies

The interaction of a GPCR with its endogenous agonist can be neutralized not only by small molecules and peptides, but also by immunological approaches. To this end, antibodies were raised against the extracellular portion of the receptor molecules (Figure 2b) or against their ligands (Figure 2c). The desired neutralizing effect can be achieved either by administering a vaccine or by direct injection of antibodies or their derivatives. Several synthetic and recombinant vaccines have been developed against GnRH and human Chorionic Gonadotropin hormone (CG) and phase I/II clinical trials documented their safety, reversibility and efficacy. Serum testosterone was reduced with consequent atrophy of the prostate, a fall in prostate specific antigen and a clinical improvement of prostate carcinoma patients, however, their launch was prevented by economical considerations [57].

A similar approach was taken to neutralize gastrin by linking its 17 N-terminal amino acids to diphtheria toxoid. The resulting immunogen, G17DT, was developed up to a large phase III trial for the treatment of advanced pancreatic cancer. The study compared the gold standard treatment gemcitabine with the combination of gemcitabine plus G17DT. No survival benefit was observed for the combination despite an earlier placebo-controlled phase II trial demonstrated an enhanced survival advantage in pancreatic cancer [56] and the same combination was advantageous in gastric cancer [58].

Antibodies against both, chemokines and chemokine receptors, were developed [59,60] aimed at preventing metastases, angiogenesis, *etc.* ROAb 12, ROAb 13, ROAb 14 and ROAb 18 are reported to inhibit CCL5/RANTES, CCL3/MIP1α and CCL4/MIP1β binding [61].

Blocking antibodies have been considered with the specific aim of preventing angiogenesis. The intent is to interfere with GPCRs signaling between cancer cells and cells part of the stromal microenvironment, including: endothelial, myeloid, circulating or local stem cells [3]. Blocking S1P with a specific antibody prevented endothelial cell migration and capillary formation, inhibited blood vessel formation and reduced the release of IL-6, IL-8 and VEGF by tumor cells [62]. Analogously, protease activated receptors respond to proteases provided by the tumor microenvironment. Factor III and VII are proteases playing a central role in the coagulation cascade. Antibodies blocking the tissue factor-VIIa-PAR2 signaling on tumor cells attenuated angiogenesis and tumor growth [63]. Humanized antibodies to CXCL8/IL-8 (ABX-IL8) were shown to inhibit melanoma tumor growth, angiogenesis and metastasis [64]. More recently the efficacy in *in vivo* models of nanobodies against CXCR4 was reported. Nanobodies are antibodies with the variable fragment directly linked to the CH2-CH3 fragment, thus lacking the light chain (VHH). Such arrangement naturally occurs in Camelidae and warrants several advantages, in terms of specificity and low inherent toxicity [65]. The nanobody ALX-0651 mobilizes hematopoietic stem cells acting as an inverse agonist that counteracts not only ligand-dependent, but also ligand-independent (spontaneous) activation of the receptor. In the case of anticancer therapies against GPCRs overexpressed and characterized by significant spontaneous activation, an inverse agonist may be more advantageous as compared to neutral antagonists. Hopefully, in the near future these passive immunity approaches will translate to human experimentation.

Finally, a potential novel approach is to utilize inflammatory chemokines linked to the antigen to promote a stronger reaction against identified tumor antigens that would otherwise be nonimmunogenics [44].

## 4. GPCRs Desensitization

**Figure 3:**
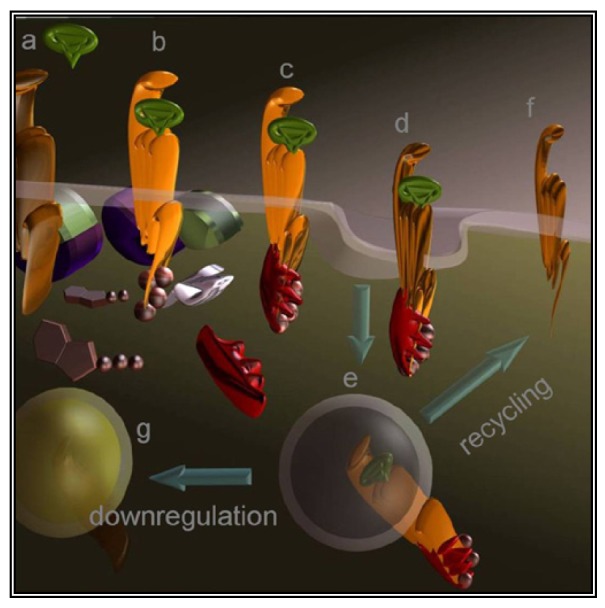
GPCRs desensitization occurs concurrently its activation. Agonist (green) binding destabilizes (**a**) the inactive state and (**b**) the receptor promotes G protein activation acting as a GTP exchange factor. At the same time, kinases (white) phosphorylate the receptor on multiple sites and (**c**) a cytosolic adaptor named arrestin (red) masks the GPCR domain binding to the G protein. Arrestin interacts with several proteins, including elements of the endosomal machinery that (**d**) recruit the GPCR to clathrin-coated pits promoting its endocytosis. (**e**) Once endosomes are formed, the GPCR can either be (**f**) dephosphorylated and return to the cell surface for another round of activation or (**g**) become proteolyzed thus reducing the number of available receptor molecules.

A less straightforward maneuver to oppose GPCRs activity is to induce their over-stimulation until natural regulatory phenomena, *i.e.*, desensitization and downregulation, will reduce signaling to background levels or below. In fact, GPCRs stimulation is paralleled by a precise sequence of events that shifts their activation threshold and lowers maximal responses. As a result, the system becomes progressively less efficient (Figure 2d and Figure 3). The molecular mechanisms underlying this effect were mostly revealed by studying opsins and the β2 adrenenoceptor. Following their activation, virtually all GPCRs become phosphorylated at multiple sites by kinases that are either activated by second messengers or by the same occupied GPCR.

Cytosolic adaptors named arrestins translocate from the cytosol to the activated GPCR and the interaction is stabilized by phosphorylation. As a result, GPCRs are uncoupled from the partner G protein and internalized thanks to endocytic machinery recruited by the same arrestin. Once in the endosomes, part of the receptors are dephosphorylated and recycled to the cell surface [66] while others are degraded by lysosomes or proteasome. Eventually, prolonged stimulation produces down-regulation leading to overall refractoriness to the stimulus and to a dramatic reduction in the number of GPCR molecules exposed at the cell surface.

Desensitization is clinically exploited by all GnRH receptor agonists (buserelin, goserelin, leuprolide, and triptorelin), however, mammalian GnRH receptors lack the C-terminus implying that the desensitization process differs from the large majority of GPCRs [67] and/or the molecular mechanisms responsible for this complex phenomenon still awaits full clarification.

A ‘desensitization based’ strategy is also being designed to target GPR54/matastin. In addition to its effects on promoting cells quiescence in response to KISS [68], GPR54 is believed to act on upstream hypothalamic neurons to increase synthesis and secretion of GnRH in specialized downstream neurons. TAK 448 is a GPR54 agonist utilized in a phase I study in prostate cancer. The intent is to desensitize GPR54 until complete cessation of signaling will determine a reduction of testosterone production [68].

S1P activates four of the five EDG-family receptors and it is believed to contribute to the etiology of cancer [17]. For this reason, approaches that do not exploit desensitization were taken to oppose S1P receptors activity. An antibody against S1P was developed at phase I, while a phase III trial is evaluating an inhibitor of sphingosine kinase (phenoxodiol) for various indications (including acute lymphoblastic leukemia, fallopian tube, ovarian and prostate cancers). The desensitization based approach was instead applied to develop FTY720, a synthetic sphingosine analog that, like sphingosine, is phosphorylated by sphingosine kinase and binds to S1P receptors. FTY720 efficiently induces internalization and polyubiquitination of S1P receptor 1 leading to its proteasomal degradation. As a result, lymphocytes become unresponsive to S1P [69]. FTY720 has been approved by FDA as the first oral treatment of multiple sclerosis, this will hopefully facilitate new trials aimed to extend the indications to cancer.

If a burst of stimulus may produce the final effect of reversing signaling, the initial stimulation clearly represents a side-effect. In the case of GnRH agonists [70], the phenomenon is known as ‘flare’ and can be minimized by co-administration of anti-androgens. Nonetheless, generally speaking, GnRH antagonists or blockers represent a better alternative.

Novel aspects concerning the usage of agonists, antagonists or antibodies, are emerging as we better understand the molecular basis of GPCRs activation. While GPCRs initially were considered as controls switching from an ‘off’ to an ‘on’ state (and *vice versa*), it has become evident that GPCRs transition through intermediate and alternative states (likely, but not necessarily, represented by steric conformations). Since differential signaling was associated to these intermediate states, optimization of the ligands with the intent of stabilizing one state as opposed to another may be crucial in the perspective of using these drugs at high concentration, and likely, for long periods of time. Furthermore, we recently described the differential sensitivity of G proteins of the G_q_ subfamily (in particular for the peculiar G15 subtype [71]) to β-arrestin desensitization [72] and the same β-arrestin emerged as versatile scaffold for important effectors upon sustained stimulation (Figure 1) [73]. GPCRs desensitization may be a process operating less uniformly than initially described and alternative signaling may take over the initial acute response. Novel treatments aiming to exploit desensitization to silence a GPCR should carefully evaluate these aspects.

## 5. Pepducins

More recently, novel molecular tools were successfully applied to modulate the tumor microenvironment in animal models of ovarian cancer with the result of preventing angiogenesis and progression [74]. These compounds, named pepducins, are N-terminally lipidated peptides corresponding to stretches of GPCRs intracellular regions (intracellular loops) [75].

Pepducins have been developed to modulate GPCRs activity from the intracellular side of the plasma membrane (Figure 2e). Protease-activated receptors (PAR1, PAR2, PAR4) and chemokines receptors (CXCR1, CXCR2, and CXCR4) are among targeted receptors that are at the same time good potential targets for anticancer treatment [74]. This approach opens novel perspectives and may shed new light on signaling events occurring intracellularly, away from the plasma membrane. Almost all natural and synthetic GPCRs ligands are hydrophobics, and historically GPCRs signaling has been interpreted as an event occurring uniquely at the plasma membrane. More recently GPCRs intracellular signaling became widely accepted although it remains very poorly characterized. GPCRs and G proteins can in fact signal also inside the cell where their presence has been documented in many subcellular compartments that include endosomal compartments, the cytosol and the nucleus [76,77]. Cytoplasmic vs. nuclear CXCR4 expression in breast cancer and NSCLC was correlated to a worse outcome and other tumor characteristics [44]. Furthermore, as briefly mentioned in the previous paragraph, GPCRs localization varies upon activation and the dynamics of GPCRs distribution may thus result instrumental to tumor signal transduction. Pepducin may reach GPCRs that are not reachable by the ligands and thus could reserve very important surprises.

## 6. Inhibitors of G Protein Signaling

As previously mentioned (end of Section 2), parallel and redundant activity of multiple GPCRs may contribute to keep active pro-cancer signaling pathways. Bypassing the GPCRs to directly target their immediate downstream interactors, would dramatically expand the spectrum of action as the signaling of hundreds of GPCRs converges onto a much smaller number of effectors. G proteins are the canonical GPCRs effectors (Figure 2f), these heterotrimers are identified by their α-subunit that for decades was erroneously considered the only signaling subunit. In humans, 16 genes codify for α-subunits and most of their products are ubiquitously distributed.

All four G protein families (G_s_, G_i_, G_q/11_, G_12/13_) have been identified as oncogenes in very specific tumoral forms [78-80]. The α subunit of heterotrimeric G proteins is distantly related to monomeric small GTPases like Ras that has long been known for its key role in cell transformation. Ras gene is mutated in 30% of human cancer. Recently, the possibility to treat cancer by targeting its acylation has gained growing attention and is being evaluated in clinical trials [81]. The α- and γ- subunits of heterotrimeric G proteins are also acylated and could be targeted by a similar strategy, yet no data supports this approach. Since very little is known about the roles that G proteins play in locations other than the plasma membrane, it is very hard to predict the outcome of an approach that would affect the subcellular localization of the proteins without necessarily inhibiting their function.

Furthermore, acting simultaneously on all G proteins appears as a poorly specific approach. Knockout mice clearly show that silencing a single G protein is generally well compensated by its close homologs while double knockouts are most of the times lethal [82]. Simultaneous inhibition of multiple G proteins appears therefore poorly tolerable by the organism. However, a direct screening based on the inhibition of G protein downstream activity, identified a lead compound (the imidazopyrazine derivative BIM-46174) that binds to all α-subunits and prevents the proper interaction of GPCRs with the heterotrimer thus inhibiting agonist-promoted GDP/GTP exchange [83,84]. BIM-46174 demonstrated anti-proliferative effects on human cancer cell lines established from different origins. In addition, by evaluating the invasive potential of transformed human colon cancer cells in collagen type-1 gels, BIM-46174 inhibited the effect of the Wnt oncogenic pathway. The combination of BIM-46174 with either a farnesyltransferase inhibitor or cisplatin reduced tumor growth rate in a mouse model based on xenografts established from human lung carcinoma cell lines. Similarly, combined treatment with BIM-46174 with a topoisomerase inhibitor showed significant anti-tumor activity against pancreatic cancer. It is encouraging and surprising that, beside a limited weight loss, there was no evidence of toxicity in a preliminary assessment, however, no pharmacokinetic profile was described for this compound and no further development was reported.

YM-254890 has been proposed as a G_q_ specific inhibitor and G_q_ has recently been shown mutated blue naevi and ocular melanoma of the uvea [79]. Neither BIM-46174, YM-254890 or related compounds have yet been tested in clinical trials.

Suramin is also considered an inhibitor of G protein signaling. Suramin is a poly-sulfonated naphthylurea, currently used to treat African river blindness and African sleeping sickness. It has also been tested in several clinical trials to treat many forms of cancer, particularly prostate cancer. However, earlier trials were not conclusive and did not control for anti-androgen withdrawal phenomenon, that at the time was yet to be described [85]. Significant toxicity was associated with effective doses preventing FDA approval. Suramin is also an antagonist for purinergic GPCRs and RTKs therefore it is unclear how directly its clinical activity relates to preventing the dissociation of the heterotrimer [86]. Clarifying these important details, improving pharmacokinetic and a more modern design of clinical trials could provide new openings in the future.

More recently, direct signaling of βγ dimers toward PI3K, Src *etc.* has been documented in the regulation of cell motility, growth and differentiation [87,88]. A specific βγ inhibitor (M119K) has been identified, but, so far, very limited efforts have been made to act pharmacologically on any of the 5 β- and 12 γ- subunits expressed by the human genome [89].

Highly debated in the past, G proteins-independent signaling is today widely accepted and alternative signaling mediators have been identified, including arrestin. Lacking enzymatic activity, arrestin acts like an adaptor which, depending on the GPCR subtype [66], may or may not follow the internalized receptor molecule to subcellular organelles dragging docked kinase and/or other proteins [73].

Specific interactions have been reported between arrestin and a plethora of signaling elements [73,90]. Arrestin accumulation in subcellular locations, including the nucleus [91], implies important consequences as it may redistribute signalosomes assembling key-players in cancer signal transduction, like mitogen-activated protein kinases, Akt, PI3 kinase [73,92], transcription factors like Gli [93] or NF-kB and its regulators [94]. For instance, PAR2-mediated recruitment of arrestin to pseudopodia promotes breast cancer cell migration and facilitates cytoskeletal reorganization [95]. No specific inhibitor of arrestin function has been reported for any of four arrestin subtypes present in our organism despite, because of their well mapped modular organization, it appears reasonable to interfere with specific functions and spare generalized effects.

## 7. Targeting Toxins and Isotopes to Cancer Cells

Viruses like HIV utilize GPCRs (CXCR4 and CCR5) as lymphocyte-specific docking sites for infection. Each mammalian cell expresses dozens of different GPCRs [96]; cancer cells are no exception deploying a collection of GPCRs that combines tissue-specific GPCRs to others abnormally expressed during the transformation process. Many investigators took advantage of the GPCR expression profile of a cancer cell to specifically deliver cytotoxic molecules such as toxins or isotopes. Prototypical molecules have been created linking toxins to the variable fragment of an antibody. The resulting chimeric molecules are termed immunotoxins (Figure 2g). Natural toxins derived from microorganisms have been typically engineered to improve their efficacy and reduce nonspecific binding. Initially, the targeting moiety have been most commonly directed against cluster of differentiation molecules (CD) to treat hematologic malignancies [97] but soon afterwards many other cell surface molecules have been selected as the approach was extended to different cancer forms.

Following the same strategy, the cargo can be opportunely delivered by exploiting the high specific interaction occurring between the GPCR and its ligand. An important advantage is that an agonist normally promotes GPCRs internalization and therefore actively accumulates the drug inside the cell. To this purpose, the drug will have obviously to be designed so that the ligand maintains its ability to induce the GPCR conformational change that triggers the interaction with arrestin followed by endocytosis. The active accumulation of the cytotoxic agent inside the cell is expected to minimize the effective dosage and thus lower side effects. This aspect could be further improved by utilizing pro-drugs that become active only within the endosomal lumen or in the following intracellular trafficking steps [98].

The use of membrane disrupting peptides linked to GPCR peptide agonists has been proposed in alternative to toxin-ligand chimeras. Various organisms evolved defense mechanisms against external attacks based on linear, positively charged, amphipatic peptides that in the plasma membrane bilayer assume an α-helical conformation and affect the electrochemical potential. Based on the naturally occurring lytic peptides, artificial peptides (Hecate, Phor14, Phor21) conjugated to the β chain of CG have been proposed for the treatment of testis, ovary, prostate and breast cancer. Animal models showed that these small peptides are rapidly metabolized and are not antigenic [99-101]. Targeting LH/CG receptors spared toxicity to other organs, although gonadal tissues still showed some changes.

Radiolabeled and, more recently, nanoparticle-linked peptides (SST, bombesin, cholecystokinin [102], *etc.*) have been used for *in vivo* imaging of inflammatory conditions and metastasis. The same reagents have been proposed for ‘peptide-receptor radionuclide therapy’ [103] to target for instance SST-receptor-positive endocrine tumors [104]. ^111^In, ^90^Y, ^177^Lu chelated to SST analogs (cyclic octapeptides) have been administered at high doses. The premises are positive, adverse effects such as radioactivity accumulation in the kidney and hematological consequences appear limited as compared to other treatments such as chemotherapy [104]. Higher doses of radioactivity could possibly reproduce in humans results in tumor remission achieved in animal models. It is clear that parallel treatments will have to be developed to protect the kidney and the bone marrow. Explorative scintigraphy would warrant that GPCRs in the tumor cells effectively uptake radiolabeled peptides.

An important advantage of all the strategies described in this paragraph is that they are not conditional to cell proliferation and would effectively strike quiescent stages.

## 8. Adjuvant Therapies

All approaches described above are intended to act on GPCRs expressed on the cancer cell, however, there is rationale to design pharmacological treatments acting also on cells that are not transformed but support the tumor. Under these circumstances, GPCRs signaling could be modulated to provide adjuvant treatment.

For example, transient vasodilatation of the blood flow to the tumor may increase the delivery of anticancer agents to the tumor mass. As compared to normal blood vessels, tumor vessels are relatively devoid of smooth muscle cells: a highly selective peptide agonist of the ET_b_R (SPI-1620) has been developed to act on vessels created by tumor angiogenesis. Currently SPI-1620 entered a phase I study as an adjunct of chemotherapy [105]. An analogous effect was observed in a model of pancreas adenocarcinoma: the treatment with Smo inhibitor transiently increased the density of the tumor vasculature and increased the concentration of gemcitabine reaching the tumor cells [106] (this despite signaling via Hh is considered pro-angiogenic).

In order to sensitize a tumor to therapy, it may result even more beneficial to drive cancer cells out from their protective compartments. CXCR4 antagonists are in use to facilitate the mobilization of hematopoietic stem cells for autologous transplantation in non-Hodgkin's lymphoma and multiple myeloma. In a murine model of acute myeloid leukemia two inhibitors of CXCR4 function (AMD3100 or AMD3465) alone or in combination with granulocyte colony-stimulating factor, enhanced anti-leukemic effects of chemotherapy by targeting leukemia/bone marrow microenvironment interactions [107,108]. It becomes now crucial to identify the signals retaining stem cells (normal or cancer) in the many microenvironments that are rapidly being characterized in various organs [109].

On the other side, being able to actively navigate selected cell types in the organism may help to focus the immune system against the tumor. Cancer vaccines aim to elicit tumor-specific adaptive immune responses. Activated T lymphocytes are expected to play an essential role in attacking residual disease after initial reduction of the bulk of the tumor or at the least to limit tumor progression. Chemokines mediate the recruitment of antigen presenting cells such as DC or macrophages and their interaction with T lymphocytes. Mice model provided evidence that chemokines like CCL2, CCL3, CCL20 opportunely administered in the tumor site promote antitumor response [110-113]. The effect is likely mediated by the recruitment of effector cells expressing CCR1/CCR5 (*i.e.,* monocytes, natural killer cells and T lymphocytes). Proof of efficacy in human is still lacking and the delivery of intratumoral chemokines remains an unsolved issues that may require more sophisticated approaches. Animal models showed that DC pulsed with the antigen elicit more effective antigen-specific immune responses if previously engineered to express CCR7. CCR7 expression directs cells toward the lymph node where DC present the antigen to lymphocytes [114]. Genetic manipulation has also been proposed in analogous circumstances to provide a source of tumor-specific T cells and thus address another major reason for the failure of adoptive immunotherapy against most common cancers. To circumvent this problem, it has been suggested to engineer lymphocytes to express GPCRs responding to the chemokines produced at the tumor site [115].

A phase I study is being carried out with ECI 301, a derivative of human CCL3 (MIP1-α) binding to CCR1 and CCR5 and likely involved in defining the composition of tumor infiltrating cells [116]. Systemic injection of ECI 301 improved the immune response after radiotherapy or radiofrequency ablation [117]. ECI 301 has been shown not only to enhance antitumor radiation efficacy but also to prevent tumor growth distant to the irradiation site (referred to as the abscopal effect). However, mouse models show that the efficacy of chemokines treatment largely depends on the antigenicity of tumor. To potentiate the therapeutic potential of chemokines it has been proposed to promote local innate immune responses with immunostimulatory chemokines or to block immunosuppressive cytokines.

The approaches described above count on the use of agonists/antagonists. The importance of suppressive populations of immune cells, typically lymphocytic and dendritic cells lineages, is emerging as a requirement to allow the cancer to escape the control of immune system. A radical approach to eliminate suppressive cell lineages could take advantage of their specific GPCRs profile to deliver cytotoxic cargo (as described in the previous Section).

## 9. Conclusions

Successfully defying a tumor today mostly counts on surgical eradication. As we better understand the molecular basis of the transformation process, pharmacological treatments will certainly become more effective. One of the greatest expectations is to be able to minimize side effects by focusing on cancer-associated signal transduction rather than on general processes like DNA synthesis [118].

Unfortunately, a common finding in new personalized pharmacological therapy is that remarkable initial responses are reversed by drug resistant mutations. For instance, Smo antagonist GDC-0449 is now in phase II on large variety of advanced cancer forms after it induced tumor regression in medulloblastoma mice models and in basal cell carcinoma patients. However, a clinical trial on a patient affected by medulloblastoma showed that the initial response to the treatment ceased after 3 months, likely because a mutation in Smo produced resistance to cyclopamine[32]. This is highly reminiscent of what observed for other signaling proteins, see for instance resistance to imatinib provoked by BCR-ABL mutations [119]. In addition to genetic instability and dynamic adaptation of tumor cells, in deadly forms of malignancies it has repeatedly been shown how chemotherapy can expand the pool of resistant CSC sreversing the initial effectiveness of chemotherapy [120]. Combining drugs to hit multiple targets may provide the strategy to move the threshold forward and prevent tumorigenic cells adaptation. Preclinical models indicate that combination of therapies may become additive/synergistic while single agents only induce growth delay [106,118,121].

RTKs amplify the mitogenic activity of ET_a_R and ET_a_R transactivates the EGF receptor (EGFR). This provides a rationale for combining EGF and ET antagonists. By antagonizing the ET_a_R, zibotentam reduced EGFR transactivation while gefitinib partially reduced ET effects reducing EGFR phosphorylation. Simultaneous blockage of these interconnected circuitries may therefore critically enhance single antitumor activities [25]. Attacking cancer from multiple fronts with personalized therapies may therefore represent the key. GPCRs can offer the flank to highly specific approaches capable of striking where conventional therapies cannot reach (*i.e.*, targeting CSCs in their niche and thus prevent relapses and resistance). Up to date, in oncology 5-6 GPCR isoforms are targeted aiming at palliative care (*i.e.*, cannabinoids receptors), adjuvant therapy or to indirectly inhibit cancer growth (see Introduction). More than one third of marketed drugs target less than fifty GPCRs leaving hundreds of potential new options, including more than a hundred orphan GPCRs, as novel opportunities for developing new anticancer agents.
